# Supplementary material for: Active site specificity profiling datasets of matrix metalloproteinases (MMPs) 1, 2, 3, 7, 8, 9, 12, 13 and 14
Source: Data Brief. 2016 Feb 22;7:299–310. doi: 10.1016/j.dib.2016.02.036 (PMC4777984; doi:10.1016/j.dib.2016.02.036)
Supplement: Supplementary file 10 — Supplementary material [file mmc10.zip › WebPICS_hMMP7_T_1%/P3prime.html]

 

PICS results


|  |  |
| --- | --- |
| **P3prime\_A**  39 in 291 sites   13.4 %    effects > 10 perc. pnts.   (vice-versa in brackets)  P3\_I: 10.5 (14.2) |  |
| **P3prime\_D**  22 in 291 sites   7.6 %    effects > 10 perc. pnts.   (vice-versa in brackets)  P2\_Q: 11.3 (12.4)   P1\_P: 14.1 (12.4) |  |
| **P3prime\_F**  14 in 291 sites   4.8 %    effects > 10 perc. pnts.   (vice-versa in brackets)  P2\_Y: 10.9 (15.2) |  |
| **P3prime\_G**  36 in 291 sites   12.4 %    effects > 10 perc. pnts.   (vice-versa in brackets)  P2\_G: 11.2 (12.6)   P1\_S: 11.2 (16.8) |  |
| **P3prime\_H**  12 in 291 sites   4.1 %    effects > 10 perc. pnts.   (vice-versa in brackets)  P1\_F: 19.5 (14.7) |  |
| **P3prime\_I**  19 in 291 sites   6.5 %    effects > 10 perc. pnts.   (vice-versa in brackets)  P2\_N: 13.2 (10.9) |  |
| **P3prime\_N**  13 in 291 sites   4.5 %    effects > 10 perc. pnts.   (vice-versa in brackets)  P2\_C: 28.4 (52.6)   P2\_M: 19.7 (25.5)   P1\_H: 12.0 (15.5)   P2prime\_M: 17.6 (14.3) |  |
| **P3prime\_Q**  20 in 291 sites   6.9 %    effects > 10 perc. pnts.   (vice-versa in brackets)  P2\_Q: 13.1 (13.1)   P1\_E: 27.3 (14.7) |  |
